# Identification and chemical profiling of anti-alcoholic liver disease biomarkers of ginseng Huang jiu using UPLC-Q-Orbitrap-HRMS and network pharmacology-based analyses

**DOI:** 10.3389/fnut.2022.978122

**Published:** 2022-08-12

**Authors:** Yongxi Wu, Yongyu Cai, Liting Ma, Fangtong Li, Meiyu Zhang, Yizhu Wang, Fei Zheng, Zifeng Pi, Hao Yue

**Affiliations:** Changchun University of Chinese Medicine, Changchun, China

**Keywords:** ginseng Huang jiu, ingredient analysis, UPLC-Q-Orbitrap-HRMS, orthogonal partial least squares-discriminant analysis, network pharmacology

## Abstract

This study investigated the mechanism of characteristic non-volatile organic compounds (NVOCs) from ginseng Huang jiu (GH) in the treatment of alcoholic liver disease through UPLC-Q-Orbitrap-HRMS and network pharmacological analyses. Changes in NVOC contents in ginseng Huang jiu and ginseng-soaked wine fermented by different processing technologies were analyzed through liquid chromatography–mass spectrometry (LC-MS). A total of 96 ginsenosides were identified in ginseng Huang jiu throughout the fermentation process, which included 37 protopanaxadiol-type ginsenosides, 47 protopanaxatriol-type ginsenosides, and 4 oleanolic acid-type ginsenosides. Orthogonal partial least squares-discriminant analysis (OPLS-DA) revealed that 20(R)-Rg2, Gypenoside XVII, 20(S)-Rf3, CK, Rg5, Rh2, and other rare ginsenosides in ginseng Huang jiu could be the potential index for determining ginseng Huang jiu. In addition, ginseng Huang jiu could improve alcoholic liver disease by regulating the GSTP1, HRAS, AKR1B1, GSTA1, Androgen receptor (AR), GSR, and LDHB genes through bioinformatics analysis. This study provides new insights into improving the industrial production of ginseng Huang jiu and treating alcoholic liver disease with medicinal and food products.

## Introduction

Huang jiu is a popular Chinese traditional alcoholic beverage with the reputation of being the “eldest of all medicines” (1). As a kind of brewed wine with low alcohol content, rich nutrients and health-care function, Huang jiu had a broad development prospect. Chinese medicinal herbs are frequently present in low-level fermented wines because of their significant homology of medicine and food, nutritional value, and tonic effect. The nutrition and flavor of Huang jiu have been enhanced by current research and development of fermented health wines in the domestic Chinese market, which has also helped extend the demand for Huang jiu.

*Panax ginseng* C. A. Mey (ginseng) is widely used as a healthy meal, nutritional supplement, and medicine (2). Ginseng is abundant in ginsenosides, which exhibit activities of anti-cancer, antioxidant, and anti-cardiovascular (3, 4). Ginsenosides are the main active ingredients and exert therapeutic effects on sleeplessness, palpitations, mood disorders, and depression (5). Fermentation can reduce the number of glycosyl groups on ginsenosides, resulting in rare ginsenosides with improved bioavailability and pharmacological action (6). Ginseng is potentially valuable in developing functional foods, dietary supplements, or nutritional products. The current applications of ginseng in food include ginseng drinks and yogurt, but ginseng Huang jiu (GH) has not yet been reported (7, 8). In low-alcoholic Huang jiu, the aroma components influence its quality assessment, whereas non-volatile components reflect its better efficacy. Various biological or chemical events occur during fermentation, including synthesizing and eliminating non-volatile organic compounds (NVOCs) (9). As a result, NVOCs play a critical role in determining the degree of GH fermentation. However, changes in the NVOC composition of GH remain unknown. Its indication components could be the difference in the compounds between GH and ginseng-soaked wine (GSW). Therefore, changes in NVOCs during ginseng fermentation must be determined to improve the quality of GH. Ginsenosides are the main non- NVOCs of GH. Therefore, this paper focuses on ginsenosides as indicators for evaluating GH.

Recent studies have shown that many fermented spirits contain biologically active compounds with antioxidant, immune-enhancing, and anti-aging activities, which may help alleviate alcoholic liver disease (ALD). For example, resveratrol has been found in rice wine and wine, which could protect the liver through various effects, such as anti-inflammatory, anti-apoptotic, and antioxidant (10, 11). Chinese Baijiu improves the structure of the intestinal flora or reduces EtOH-induced liver damage in the action of intestinal flora (12). Not coincidentally, malic and citric acids produced in millet Huang jiu also exert hepatoprotective and anti-fatigue effects (13). Therefore, we hypothesize that the non-volatile fermented metabolite ginsenosides in GH have hepatoprotective effects in chronic alcoholism and that ingestion of GH improves ALD.

This study aimed to investigate variations in the composition of NVOCs in different fermentation processes of ginseng wine through orthogonal partial least squares-discriminant analysis (OPLS-DA) in conjunction with UPLC-Q-Orbitrap-HRMS. Under controlled laboratory conditions, a multivariate analysis was performed to create an index for predicting the putative active components of GH. To gain further insights into the molecular mechanism of GH in the treatment of ALD, network pharmacology was performed for the predictive screening of the targets and visual analysis was used to explore the relationship between GH, gene targets and pathways. The results will provide a theoretical basis for the application and quality control of GH.

## Materials and methods

### Chemicals

Ginseng root was obtained from Wanliang County, Fusong City, Jilin Province, China and identified by Prof. Shumin Wang of the Changchun University of Chinese Medicine. Angel wine yeast and yellow rice were purchased from a local supermarket. *Saccharomyces cerevisiae* was purchased from France Lesaffre Danbaoli Yeast Co., Ltd. Acetonitrile, formic acid, and methanol from American Tedia Reagent Co., Ltd. Ultrapure water was prepared with a MillQ system by the Millipore Co., of USA. Ginsenoside Rb1, Rd, Rc, Rb3, Re, CK, 20(S)Rg2, 20(R)Rg2, 20-*O*-Glc-Rf, Gypenoside XVII, and standard chemicals were purchased from Shanghai Standard Technology Co., Ltd. (purity ≥ 98%, Shanghai, China).

### Fermentation processing technology of GH and GSW

In GH preparation, the optimal main fermentation process was obtained through response surface analysis under the following conditions: ginseng addition of 8 g, material-to-liquid ratio of 1:4 g/mL, fermentation time of 8 days, fermentation temperature of 28°C, yeast addition of 0.7%, and alcoholic strength of 8%. The conditions for post-fermentation are shown below: post-fermentation time of 7 days and post-fermentation temperature of 16°C. Immediately after the post-fermentation, we collected samples. Obtained samples were centrifuged (3,000 rpm, 20 min), and the supernatant was analyzed.

The GSW process is as follows. Cut the ginseng into small slices and soak them in the Huang jiu we have previously configured. Then, the alcohol was adjusted to 8°. After the same time of incubation as above for GH, the GSW preparation was completed, and samples of GSW were then collected. Obtained samples were centrifuged (3,000 rpm, 20 min), and the supernatant was analyzed. All samples were evaluated in six copies.

### Preparation of samples for analysis

To obtain a sample solution, 50 mL of GH or GSW was added with an equal amount of water-saturated n-butanol for extraction four times. The solvent was evaporated in a water bath at 40°C. The dried extracts were weighed accurately and redissolved in methanol to 10 mL for further analyses. The extractions of each sample were preserved at −20°C. The redissolved methanolic solution was filtered with a 0.22 μm filter before injecting into the UPLC-Q-Orbitrap-HRMS system.

### UPLC-Q-Orbitrap-HRMS analysis

The chemical constituents of ginseng were analyzed on a Thermo Dionex Ultimate 3000 UPLC system equipped with a binary pump, degasser, autosampler, and a column compartment (Thermo Fisher, San Jose, CA, United States). The sample was separated on a Sigma HPLC Column C18 (50 mm × 3.0 mm × 2.7 μm; Massachusetts, United States) eluted with a mixture of 0.1% formic acid (A) and acetonitrile (B). The proportion of acetonitrile (B) was increased from 5% (0–10 min), 5–10% (10–15 min), 10–15% (15–18 min), 15–20% (18–20 min), 20–25% (20–21 min), 25–30% (21–22 min), 30–35% (22–25 min), 35–40% (25–30 min), 40–60% (30–35 min), 60–80% (35–38 min), 80–100% (38–40 min), 100–100% (40–45 min), and finally adjusted from 100 to 5% (45–50 min). The flow rate was 0.4 mL min^−1^, and the column temperature was set at 30°C. The injection volume was 5 μL.

Mass detection was performed on a Q-Exactive Orbitrap Mass Spectrometer (Thermo Fisher, San Jose, CA, United States) equipped with an Electron Spray Ionization source in negative ion mode. Mass conditions were set: sheath gas (N_2_) flow rate of 4 × 10^6^ Pa, drying gas temperature of 350°C, and auxiliary gas temperature of 300°C. The full-scan mass spectrum was recorded in *m/z* 150–2,000 Da at seven spectra/s. MS/MS experiments were set as data-dependent scans. The MS/MS data were acquired in Full-MS/ddMS2 mode under the following settings: a resolution of 17,000 with an automatic gain control target of 1 × 10^5^, a maximum injection time of 50 ms, and normalized collision energy of 25–55. Thermo Xcalibur controlled all data acquisitions.

### Potential mechanism of GH in the treatment of ALD using network pharmacology

#### Collection of ingredients in GH

The metabolites with VIP values >2 (*p* < 0.05) were selected as GH's differential metabolites by the OPLS-DA model. Targets of ingredients in GH were obtained by entering the SMILES numbers of each ingredient into the Swiss Target Prediction basement, which predicted targets of the ingredients based on their 2D and 3D structures (14).

#### Construction of network and bioinformatics analysis

PharmMapper (http://www.lilab-ecust.cn/pharmmapper/) databases were used to find potential targets for GH components. Gene Expression Omnibus GSE28619 mRNA microarray datasets were analyzed to find differentially expressed genes (DEGs) between ALD and GH. The GeneCards databases were searched for disease targets relevant to ALD. Uniprot (https://www.uniprot.org/) changed the target protein names to official gene names. A Venn diagram of the components and disease targets was prepared, and the intersectional targets were considered potential therapeutic targets of GH in ALD. The potential targets for GO and KEGG enrichment analyses were analyzed using the R-packet ClusterProfiler. The protein–protein interaction (PPI) network was created by entering the ingredient–disease targets into STRING. The GH–ingredients–disease targets network model was constructed using Cytoscape to show the link between ingredients and disease targets.

### Data processing and statistical analyses

The datasets were saved as.csv format files and then imported into SIMCA-P software (version 16.0, Umetrics, Sweden) for multivariate statistical analysis. Then, models of PCA and partial least squares-discriminant analysis were established. In the established partial least squares-discriminant analysis model, the compounds with VIP values >2 were marked and screened by a *T*-test (SPSS19.0, USA). Compounds with *p*-values <0.05 were considered significant contributions to the grouping and were selected as potential markers. All data were expressed as mean ± SD from three independent experiments. Differences were analyzed using a one-way analysis of variance (ANOVA) followed by a *post-hoc* test (Tukey) using SPSS 20. Statistical significance was considered at *p* ≤ 0.05. Total ion chromatography was mapped for UPLC-Q-Orbitrap-HRMS using Origin 8.0 software. TB tools 1.0 software was used to produce a clustering heat map (15). Hotelling's t-squared distribution was used to determine statistically significant differences among groups; outlier samples of the ellipse region that defined the modeled variation with a 95% confidence interval were excluded from further analyses.

## Results and discussion

### Analysis of non-volatile compounds by UPLC-Q-Orbitrap-HRMS

A UPLC-Q-Orbitrap-HRMS approach with a FULL SCAN mode was employed to characterize the non-aroma characteristics of ginsenosides of GH and GSW. For full-scan experiments, the obtained raw data were preprocessed using Sieve software (version 2.1, Thermo Fisher, USA), which extracted compound masses, retention times, and peak areas from total ion current data and other information. The parameters of Sieve software were set as follows: When performing peak matching correction, the maximum retention time deviation was set to 0.25 min, the maximum mass-to-charge ratio deviation was set to 10 ppm, and the minimum signal intensity of peak extraction was 10^6^. After the peak matching correction was completed, the total ion current of each sample was set. Each peak in the figure was normalized to the overall situation. The resulting full-scan data set included compound *m/z* @ retention time, normalized peak area, and sample number information.

**Figure 1:**
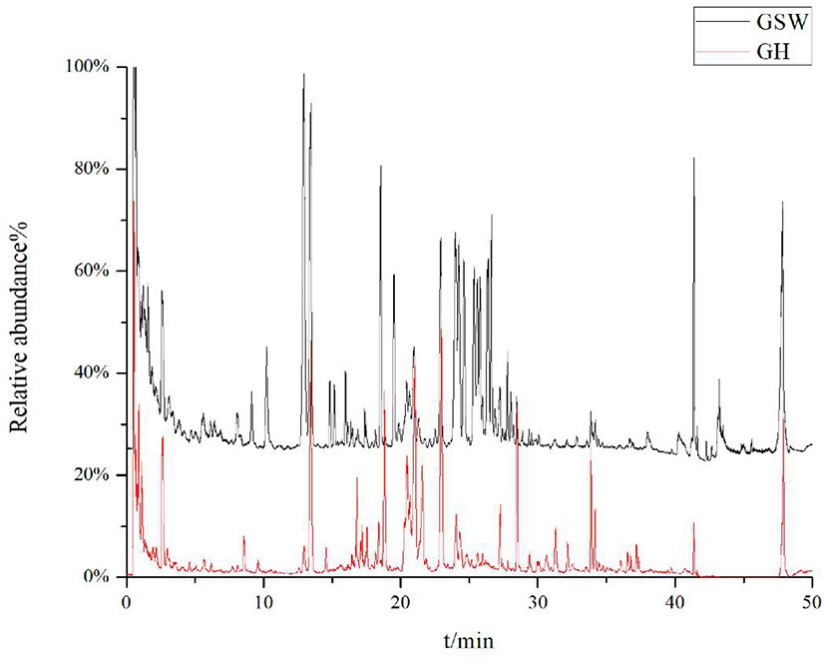
Representative UPLC-Q-Orbitrap-HRMS total ion chromatograph of ginseng Huang jiu and ginseng-soaked Wine.

The total ion chromatograms of GH and GSW are shown in Figure 1. After dereplicating the above data, constituents were initially identified by the combined method described above with an error of <10 ppm. 96 and 78 compounds were identified from GH and GSW, respectively (Supplementary Table 1). The tentative identification of compounds was conducted by comparing retention times to external standards and MS data. The most abundant compounds detected in GH and GSW were terpenoids (monoterpenes, diterpenes, and triterpenes). These ginsenosides could be divided into three classes: protopanaxadiol, protopanaxatriol, and oleanolic acids.

#### Protopanaxadiol (PPD)-type ginsenosides

**Figure 2:**
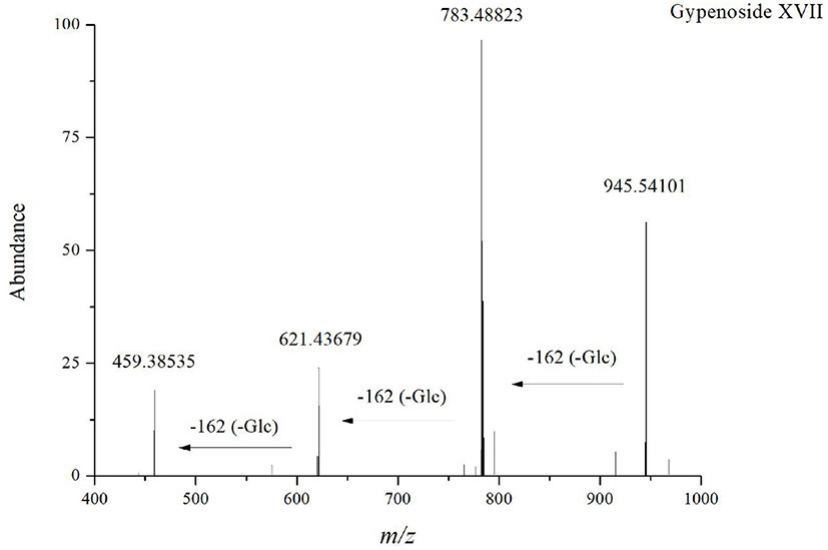
Tandem mass spectrum of ginsenosides in the negative ion mode: Gypenoside XVII.

**Figure 3:**
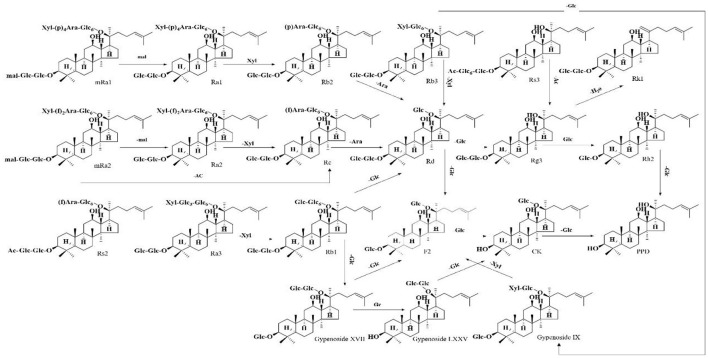
Possible transformation pathways of PPD-type ginsenosides under the action of GH.

Compound 63 (t_R_ = 28.47 min) was deduced from the high-resolution data with the molecular formula C_48_H_82_O_18_ (*m/z* 991.5483). It generated a precursor ion [M+HCOO]^−^ and fragments ions at *m/z* 945.54101 [M-H]^−^, *m/z* 783.48823 [M-Glc-H]^−^, *m/z* 621.43679 [M-2Glc-H]^−^, and *m/z* 459.38535 [PPD-H]^−^, indicating that it is a PPD-type ginsenoside containing three glucose units. Therefore, it was identified as Gypenoside XVII because of the presence of two glucose units directly linked to the low mass end at *m/z* 221.0745 and compared to standards collected under the same conditions (Figure 2). A total of 37 PPD-type *Panax* ginsenosides were discovered. The conversion pathways of PPD-type ginsenosides are shown in Figure 3.

#### Protopanaxatriol (PPT)-type ginsenosides

**Figure 4:**
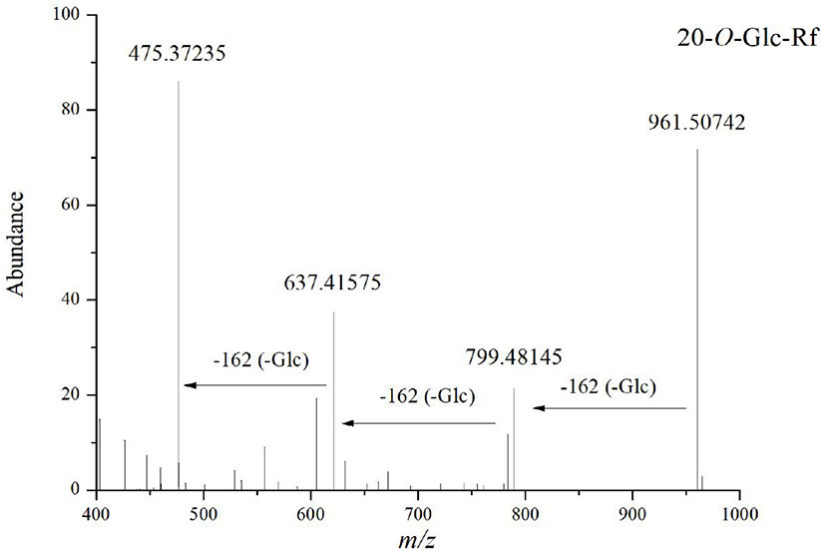
Tandem mass spectrum of ginsenosides in the negative ion mode: 20-*O*-Glc-Rf.

**Figure 5:**
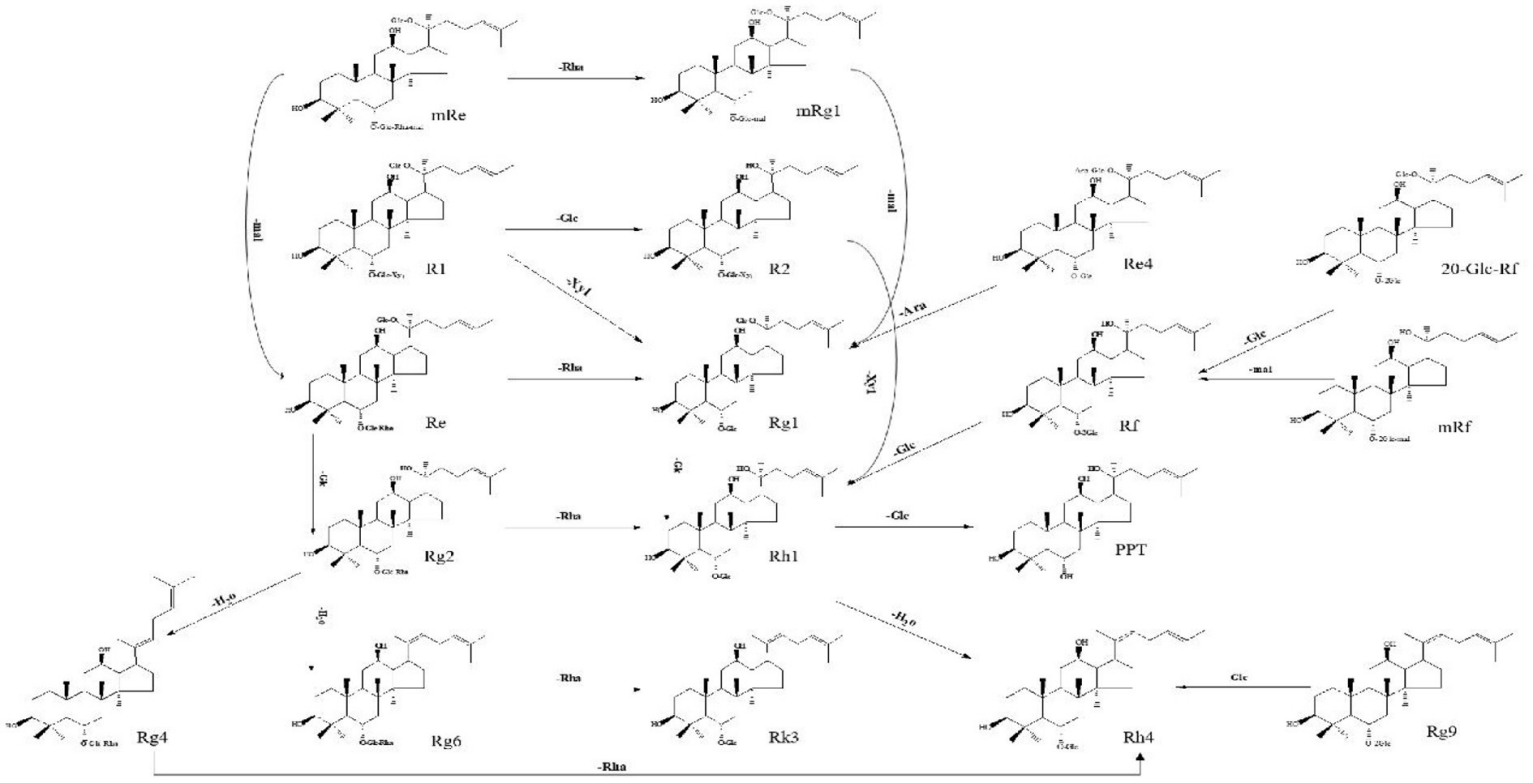
Possible transformation pathways of PPT-type ginsenosides under the action of GH.

Compound 6 (t_R_ = 5.65 min) has the molecular formula C_48_H_82_O_19_ (*m/z* 1007.5433) inferred from the high-resolution data. Cleavage of its [M+HCOO]^−^ precursor ion lost fragments at *m/z* 961.50742 [M-H]^−^ and fragments stripped of three glucose units at *m/z* 799.48145[M-H-Glc]^−^, *m/z* 637.41575 [M-H-2Glc]^−^, and aglycon ion at *m/z*475.37235 [PPT-H]^−^, indicating that it is a PPT-type ginsenoside containing three glucose PPT-type ginsenosides. Thus, it was identified as PPT-3Glc and characterized as 20-*O*-Glc-Rf *via* comparative analysis with standards collected under the same conditions. The compound was identified as 20-*O*-Glc-Rf based on the precise molecular weight, the mode of cleavage, and relevant literature (Figure 4). A total of 47 PPT-type ginsenosides were identified. The conversion pathways of PPT-type ginsenosides are shown in Figure 5.

#### Oleanolic Acid (OLE)-type ginsenosides

**Figure 6:**
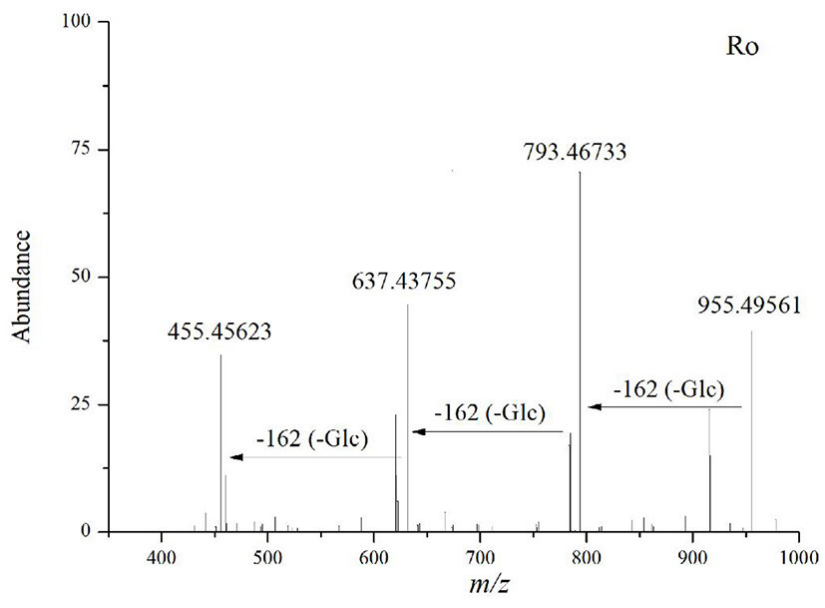
Tandem mass spectrum of ginsenosides in the negative ion mode: Ro.

**Figure 7:**
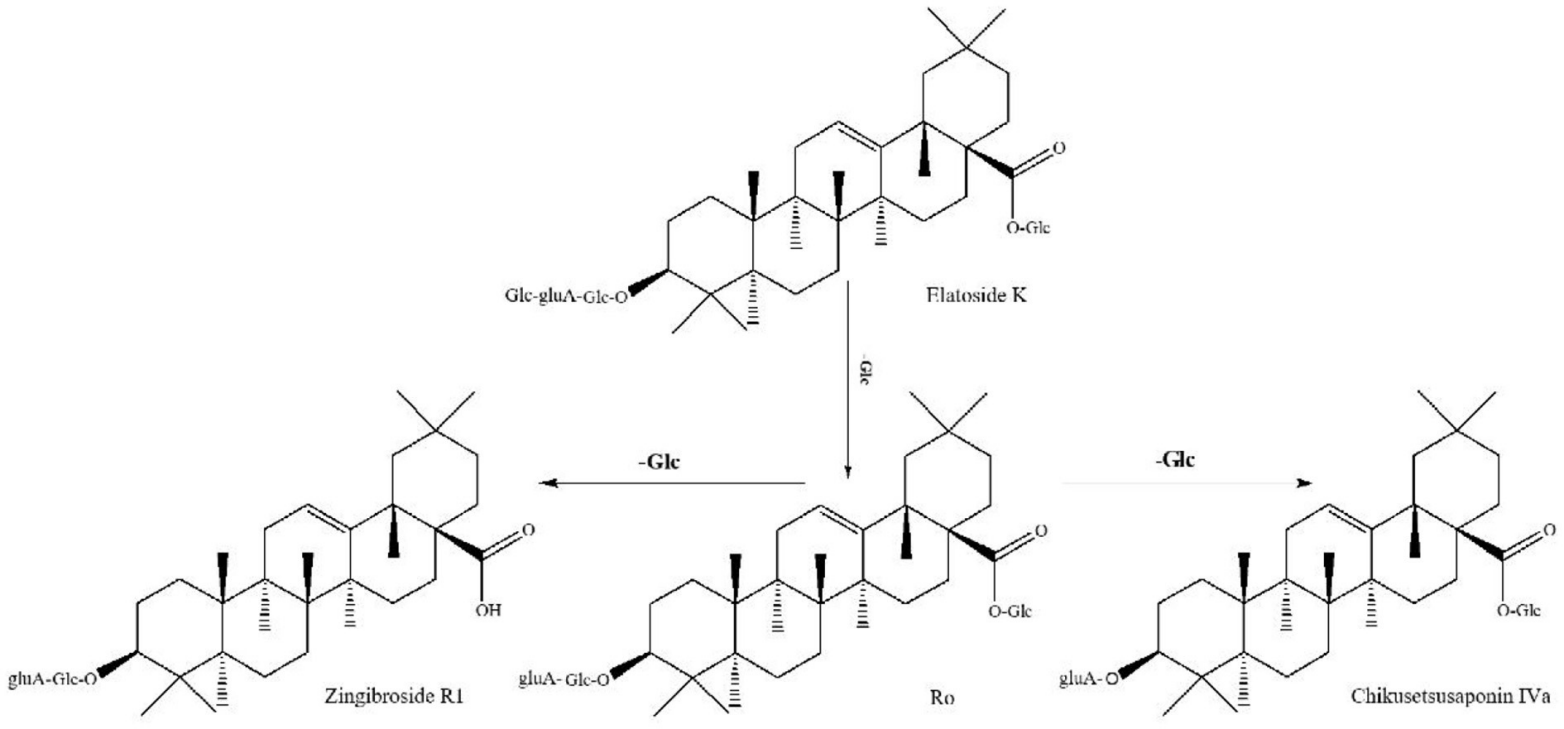
Possible transformation pathways of OLE-type ginsenosides under the action of GH.

Compound 46 (t_R_ = 23.98 min) has the molecular formula C_48_H_76_O_19_ (*m/z* 955.4956), as inferred from the high-resolution data. Cleavage of its excimer precursor ion at *m/z* 955.49561 [M-H]^−^ yielded fragments ions at *m/z* 793.46733 [M-H-Glc]^−^, *m/z* 637.43755 [M-H-2Glc]^−^, and *m/z* 455.45623 [OLE-H]^−^, indicating that it is an OLE-type ginsenoside containing two glucose units and one glucuronide unit. Therefore, it was identified as OLE-Glo and characterized as Ro *via* comparative analysis with standards collected under the same conditions (Figure 6). 4 OLE-type ginsenosides were identified. The conversion pathways of PPT-type ginsenosides are shown in Figure 7.

This study is the first to report a non-volatile ginsenoside component of GH or GSW and summarize the possible conversion pathways of ginsenosides under alcoholic fermentation conditions. Ginseng triterpenes underwent significant biotransformation under fermentation. The transformation pathways show that deglycosylation hydrolysis is the most important mode of ginsenosides transformation in the fermentation broth (16). Most rare ginsenosides were significantly increased after conversion and showed good pharmacological activity. Gypenosides XVII and LXXV in GH were identified for the first time in this study, along with relatively high levels of these two substances. Gypenosides XVII and LXXV are PPD-type ginsenosides. Gypenoside XVII was inferred to be formed by the conversion of Rb1, followed by the successive removal of one molecule of glucose to form F2, CK, and PPD. Gypenoside LXXV was formed by converting Gypenoside XVII, followed by removing one molecule of glucose to form CK. The conversion levels of Gypenosides XVII and LXXV may be influenced by various factors, such as fermentation time, pH, and the aging environment. Enzymes and microorganisms can further transform ginsenosides. We found the metabolic end products similar to Zhang's study, although PPD ginsenosides are still mostly in the form of ginsenosides CK and Rh2 (17).

### Differences in ginsenosides characteristics by multivariate data analysis

**Figure 8:**
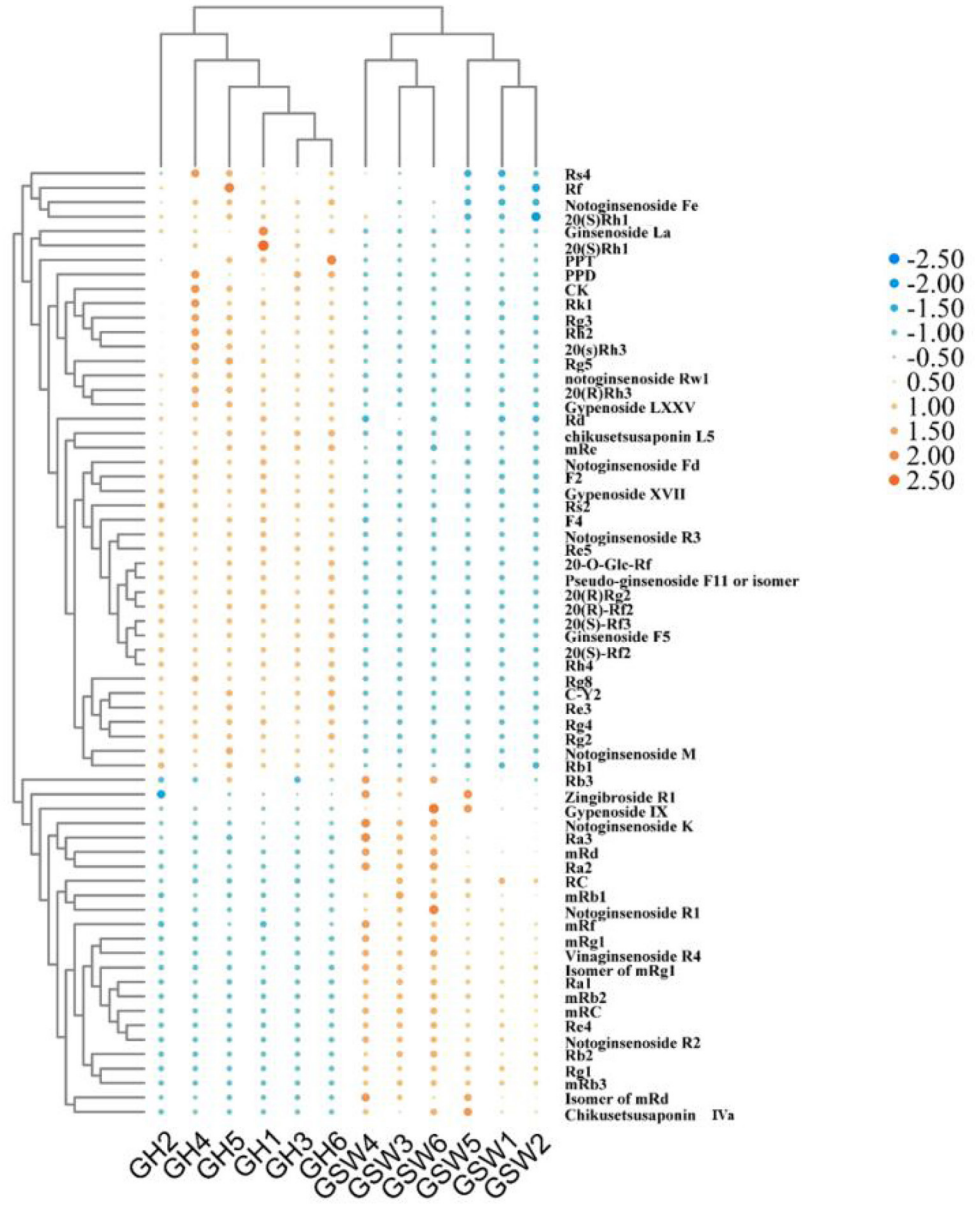
Heat map and HCA clustering results of 67 non-volatile compounds with a significant difference (*p* < 0.05) and aroma descriptors in ginseng wine samples fermented with different processes.

For mining the data obtained by UPLC-Q-Orbitrap-HRMS, single-factor ANOVA was used to analyze the 96 non-volatile components. The relationship between different processing technologies of ginseng wine samples and the 67 non-volatile components (*p* < 0.05) with important constituents was visualized by heatmap and hierarchical cluster analyses (HCA), as shown in Figure 8.

Novel rare ginsenosides, such as Rh1, F1, Rh3, Rg8, Rg9, and Rk3, were produced in GH compared with GSW. The contents of the original rare ginsenosides, such as CK, Rb1, and Rd, also increased, with the content of CK increasing by 67.83%. Ginsenosides typically contain multiple glycosyl groups, which are hydrolyzed by brewing bacteria's glycosidases to produce the corresponding deglycosylated products. The content of CK increased because it has the most extensive conversion pathway and is rich in hydrolytic enzymes and prototype ginsenosides. Because rare ginsenosides are derive from common ginsenosides, the contents of Rg1, Ra1, Ra2, and Rb3 decreased by 865.625, 513.5, 950, and 123.23%, respectively. Malonyl ginsenoside Ra2 (mRa2), malonyl ginsenoside Rg1 (mRg1), malonyl ginsenoside Rb3 (mRb3), malonyl ginsenoside Rc (mRc), and other propionylated ginsenosides almost vanished. The most significant rise in GH was analyzed in 20(R)Rg2, which increased by 299.33% when compared with GSW, and in 20(S)-Rf3, which increased by 155.67%. mRb3 and mRc had the highest decreases of 13,461.54 and 13,146.67%.

**Figure 9:**
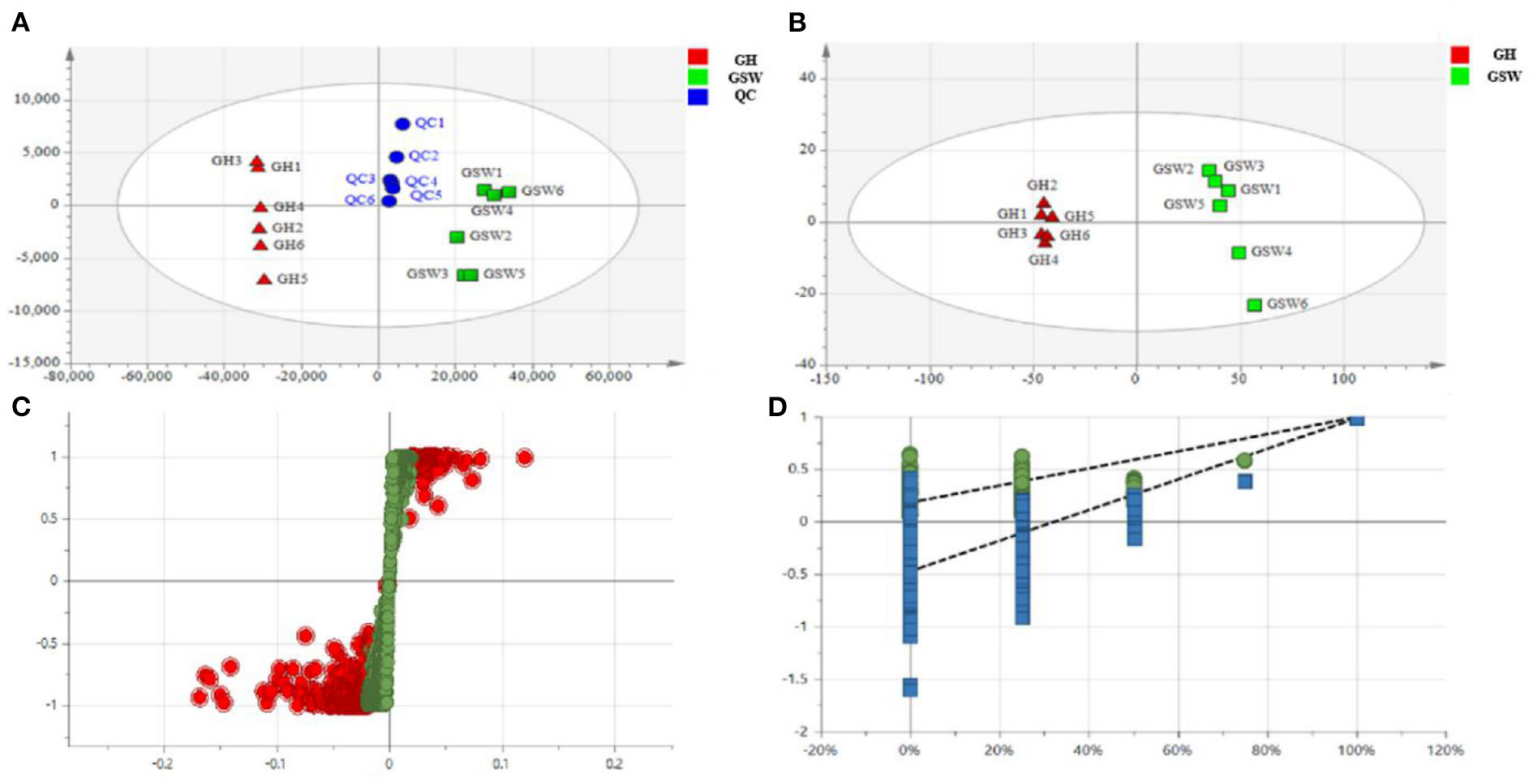
Multivariate statistical analysis of UPLC-Q-Orbitrap-HRMS metabolic profiling data. Principal component analysis (PCA) score plot **(A)**, pair-wise orthogonal projections to latent structure discriminant analysis (OPLS-DA) score plot **(B)**, and OPLS-DA/permutation test/S-plot **(C)** derived from the UPLC-Q-Orbitrap-HRMS spectra of both groups of ginseng Huang jiu (GH1–GH6, red triangles) and ginseng-soaked wine (GSW1–GSW6, green square) in negative ion mode. Statistical validation of the OPLS-DA model by permutation testing **(D)**.

The regression model between the fermentation conditions (Y-variable, *n* = 2) and the non-volatile compounds (X-variables, *n* = 96) was established *via* PCA and orthogonal partial least squares-discriminant analysis (OPLS-DA) to clarify the difference in NVOC characteristics between GH and GSW. PCA showed that the GH group were discriminated from the GSW group (R^2^X(cum) = 0.931, Q^2^ = 0.831) (Figure 9A). Furthermore, OPLS-DA loading plots revealed that the model was efficient and showed a clear separation between GH and GSW (R^2^X = 0.888, R^2^Y = 0.997, and Q^2^ = 0.993) (Figure 9B). The S-plot of GH and GSW in negative ion mode is shown in Figure 9C. Validation of the OPLS-DA model was conducted by a permutation test (Figure 9D). Overall, statistical analysis of the PCA and OPLS-DA models (R^2^X, R^2^Y, and Q^2^) demonstrated that both models were valid, with Q^2^ values close to 1 (0.831 and 0.993, respectively). As theoretical values of 1.0 would indicate a stable PCA and/or OPLS-DA model, it can conclude that the models are robust and show predictive reliability.

In addition, VIP value was used to identify the most prominent variables. In the present study, variables with VIP value >2 (*p* < 0.05) were considered to exert a significant effect on the discrimination of GH samples. A total of 21 non-volatile components could function as indicators of GH maturity during fermentation (VIP >2, and *p* < 0.05). The heat map results were similarly consistent with the OPLS-DA model, demonstrating that the latter had strong adaptability and predictability. Information on the 21 differential ginsenosides is shown in Table 1. In recent years, studies have reported that Gypenoside XVII can exert anti-acute alcoholic liver injury effects in mice by activating the PI3K/Akt and Nrf2/NF-κB signaling pathways. Gypenoside LXXV has been used to treat non-alcoholic steatohepatitis and has protective effects against oxidative stress (18). Ginsenoside Rg2 could improve high-fat-diet-induced NAFLD, and its mechanism of action is related to the dependent upregulation of SIRT1 activity (19).

**Table 1:** Basic information on the 21 active components in GH.

**No**.	**RT (min)**	**Compound**	**[M-H]-/** **[M+COOH]-** **Measured m/z**	**Formula**	**MS/MS fragment ions**	**Classification**
1.	2.42	Re3	1007.54361	C_48_H_82_O_19_	799.48225, 637.42937, 475.37986	PPT
2.	5.65	20-*O*-Glc-Rf	1007.54330	C_48_H_82_O_19_	961.50742,799.48145,637.41575,475.37235	PPT
3.	8.56	20(S)-Rf2	801.49990	C_42_H_74_O_14_	637.40578, 475.37805	PPT
4.	18.60	Rf	845.49041	C_42_H_72_O_14_	799.48234, 637.43059, 475.38017	PPT
5.	20.93	20(S)Rh1	683.43731	C_36_H_62_O_9_	475.37545	PPT
6.	21.04	20(S)Rg2	829.49711	C_42_H_72_O_13_	637.42923, 475.37849	PPT
7.	23.00	Rb1	1153.60014	C_54_H_92_O_23_	1107.59561,945.53787,783.43742, 621.37551, 459.36520	PPD
8.	26.00	Rb3	1123.59127	C_53_H_90_O_22_	1077.58161,945.54087,783.43512, 621.36482, 459.37230	PPD
9.	27.28	Rd	991.54863	C_48_H_82_O_18_	783.4174, 621.3566, 459.3527	PPD
10.	28.47	Gypenoside XVII	991.54833	C_48_H_82_O_18_	945.54101,783.48823,621.43679,459.38535	PPD
11.	31.29	F4	811.48546	C_42_H_70_O_12_	545.03567, 432.87053, 304.84772	PPT
12.	31.99	Rh4	665.42758	C_36_H_60_O_8_	545.05137, 432.90012, 304.87872	PPT
13.	32.18	F2	829.49573	C_42_H_72_O_13_	621.42669, 459.3094	PPD
14.	33.31	Rg8	827.48143	C_42_H_70_O_13_	637.43765, 475.35224	PPT
15.	33.91	Gypenoside LXXV	829.49573	C_42_H_72_O_13_	783.48256,621.43811,459.47407	PPD
16.	34.19	Rg3	829.49681	C_42_H_72_O_13_	783.47536,621.44161,459.47730	PPD
17.	36.56	Rk1	811.48512	C_42_H_70_O_12_	637.42498, 475.38021	PPD
18.	36.79	Rg5	811.48530	C_42_H_70_O_12_	765.46792, 649.93580, 545.04775, 432.89124, 304.90873	PPD
19.	37.19	Rh2	667.44291	C_36_H_62_O_8_	631.38365, 455.53292, 304.91538	PPD
20.	37.34	CK	667.44303	C_36_H_62_O_8_	621.41453,459.38370	PPD
21.	41.17	PPT	521.38212	C_30_H_52_O_4_	475.38231	PPT

### Network pharmacology to study the potential mechanism of GH in treatment of ALD

#### Collection of chemical ingredients from GH and prediction of potential targets

The potential targets of these ginsenoside components were predicted by screening GH chemical components by VIP > 2 values using the Swiss Target Prediction, and 304 potential targets were collected. Genecards were used to search for ALD targets. Using ALD as a keyword, 8,147 targets were obtained.

#### Screening of candidate genes associated with stage progression of ALD

**Figure 10:**
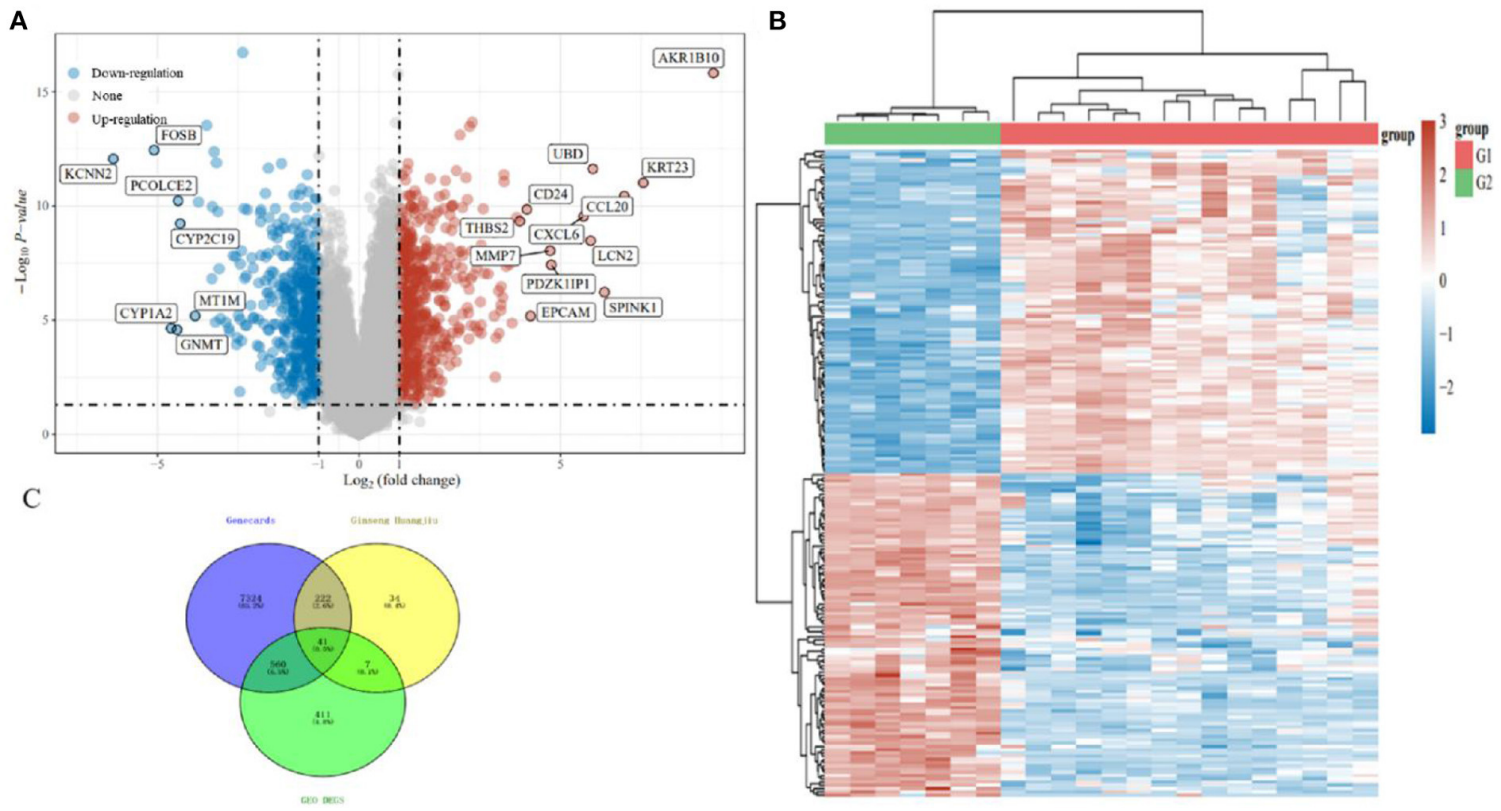
**(A)** A volcano plot was constructed using the fold change values and P-adjust. Red and blue dots indicate upregulated and downregulated genes, respectively. **(B)** Heatmap of the differential gene expression. Different colors represent the trend of gene expression in different tissues. The top 50 upregulated and top 50 downregulated genes are shown in **(C)**: intersected mRNAs from GeneCards databases, ginseng Huang jiu, and ALD.

Differential expression analysis was performed on the GSE28619 dataset to identify the key genes involved in the stage progression of ALD. As described in Section 2.5.2, all cases of GSE28619 were divided into a “Nor” group and an “ALD” group. Several DEGs between the “Nor” and “ALD” groups were discovered, as shown in Figure 10A. 548 and 471 DEGs were significantly upregulated and downregulated in the ALD samples compared with the normal samples, respectively. This study aims to find those genes associated with the stage progression of ALD. Figure 10B shows a heat map of gene expression. As shown in Figure 10C, the components and disease targets intersected, and the intersectional targets were considered potential therapeutic targets of GH in ALD. A total of 41 DEGs were finally identified, and they were defined as candidate genes and selected for subsequent analyses.

#### Prediction of common target pathways

**Figure 11:**
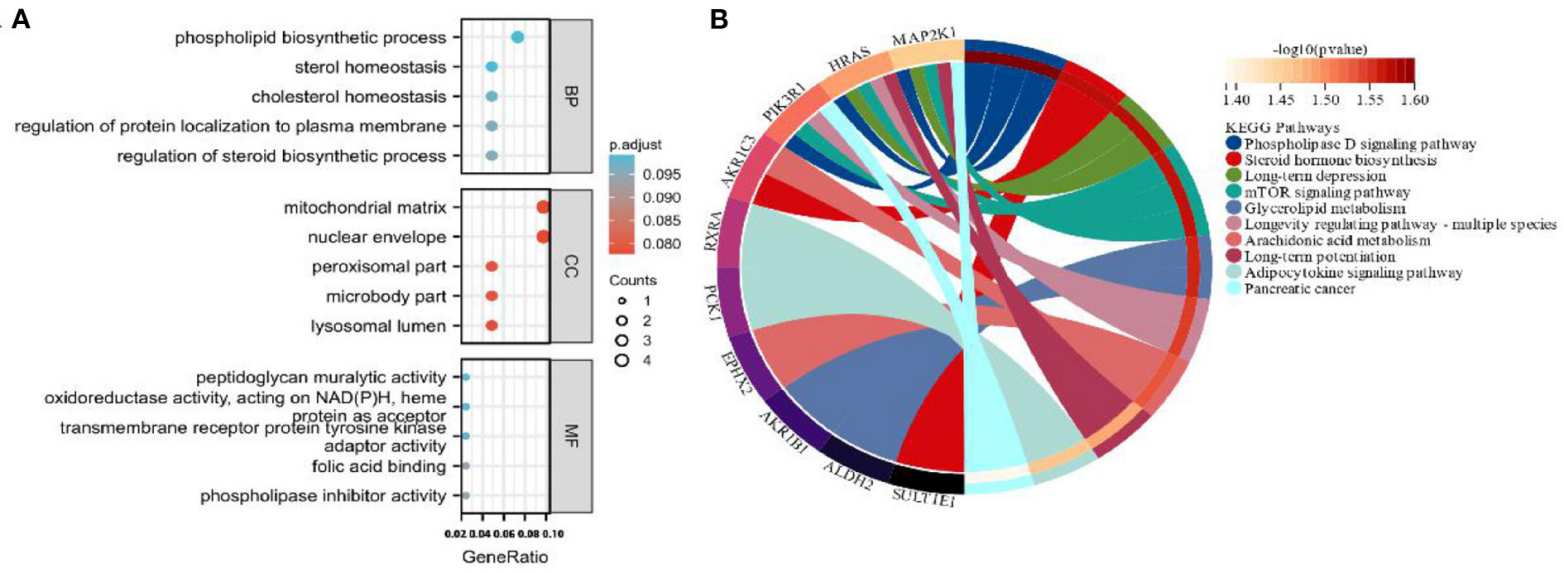
**(A,B)** GO and KEGG enrichment analyses for DEGs, terms with *p* and *q* < 0.05 were believed to be enriched significantly.

GO function enrichment analysis was performed to yield 344 BP entries, 27 cell composition entries, and 69 molecular functions (MF) entries (Figure 11A). The top five entries are plotted in order of *p-*value, where larger dots indicate a higher number of enriched genes and redder colors indicate closer GH intervention in the disease. The main areas of interest are phospholipid biosynthetic process, sterol homeostasis, cholesterol homeostasis, regulation of protein localization to the plasma membrane, regulation of steroid biosynthetic process, mitochondrial matrix, nuclear envelope, peroxisomal part, microbody part, lysosomal lumen, peptidoglycan muralytic activity, oxidoreductase activity, acting on NAD(P)H, hemeprotein as acceptor, transmembrane receptor protein tyrosine kinase adaptor activity, folic acid binding, phospholipase inhibitor activity, and other MFs and BPs. KEGG pathway enrichment analysis of key targets yielded 77 signaling pathways, with the top 10 plotted in order of *p*-value, mainly involving phospholipase D, TNF, mTOR, and adipocytokine pathways (Figure 11B).

#### Collection of ingredient–disease targets and analysis of PPI network

**Figure 12:**
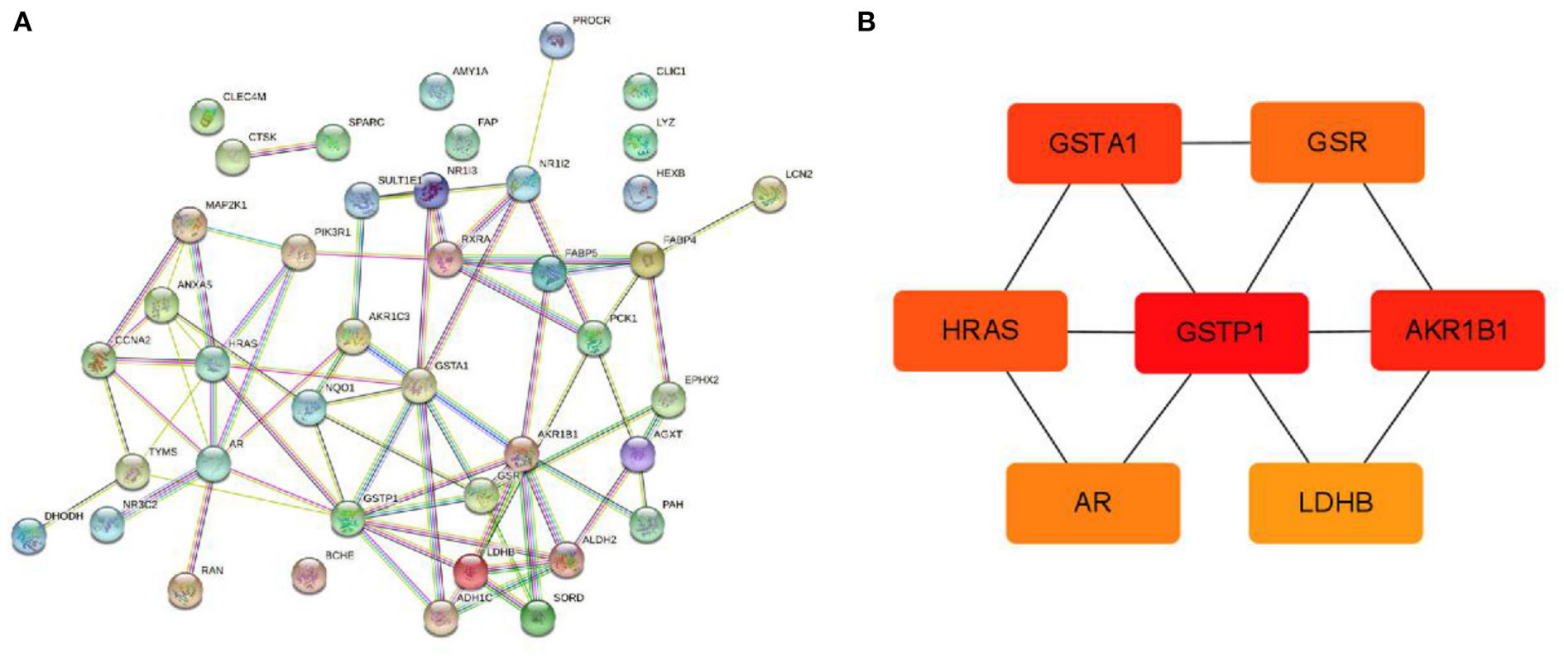
Identification of hub genes from common DEGs. **(A)** The STRING database constructed a PPI network of the common DEGs. **(B)** Seven hub genes were identified by the Cytohubba tool in Cytoscape.

According to the method, 41 ingredient–disease targets were obtained. After that, the STRING database was used to obtain a PPI network of 41 interactive targets by limiting the highest degree (degree ≥ 0.4) of confidence level and eliminating independent target proteins. The network contained 41 nodes and 71 edges (Figure 12A), in which nodes represent the target, edges represent the relationship between the targets, and different colors represent different cluster interactions. After visualization of the PPI network by Cytoscape software, hub gene screening was performed using the Cytohubba plugin. The top seven ranked key gene intersections were extracted based on MCC, DEGREE, EPC, EcCentricity, Closeness, and Betweenness scores, namely, GSTP1, HRAS, AKR1B1, GSTA1, Androgen receptor (AR), GSR, and LDHB (Figure 12B) (Supplementary Table 2).

GSTP1 is expressed in kidney and liver tissues. When the rs1695 locus encoding GSTP1 is mutated, GSTP1 will lose its enzymatic activity, allowing toxic intermediates of metabolism to accumulate, leading to the development of liver injury (20). GSTP1 can act through the Keap1-Nrf2/ARE signaling pathway and has multiple biological activities, such as antioxidation, gene regulation, apoptosis inhibition, and signal transduction (21). GSTA1 is also a member of the glutathione S-transferase family. The results of our study are consistent with those of Chang that GSTA1 can be used as an early diagnostic indicator of ALD (22). GSR is an important antioxidant enzyme class in the body. Its main physiological function is to reduce oxidized glutathione to GSH by reducing coenzyme II, thus providing reducing power for the scavenging of reactive oxygen species and protecting the body from damage (23). The anti-ALD effect of GH may be related to its capacity to protect against antioxidative stress.

HRAS is a member of the RAS gene family. Mutations in the RAS gene are associated with various tumors, with mutations in HRAS being more common in hepatocellular carcinoma, thyroid cancer, and bladder cancer (24–26). The enrichment by KEGG and the screening of hub genes suggested that GH prevents ALD from further evolving into liver cancer by regulating HRAS. The PI3K/AKT signaling pathway plays an active role in the development and progression of ALD. AKR1B1 is a major source of reactive oxygen species production and is strongly associated with hepatocellular carcinoma. Laffin showed that AKR1B1 overexpression is associated with short survival in patients with acute myeloid leukemia and multiple myeloma (27). Wang et al. similarly demonstrated that aldose reductase enhances fructose and its metabolites, providing a strong case for improving ALD (28). These results indicate that AKR1B1 could be used as a biomarker for ALD.

AR expression is strongly associated with obesity, hyperlipidemia, and fatty liver development. The incidence of hepatocellular carcinoma is significantly higher in men than in women (29, 30). AR regulates genes and pathways involved in biological processes, such as tumor angiogenesis, immune escape, infection, cell proliferation, apoptosis, and invasion, which are closely related to the development of hepatocellular carcinoma. This result confirms that GH may prevent further progression to Hepatocellular carcinoma (HCC) by improving ALD. Chen noted that LDHB expression is significantly upregulated in hepatocellular carcinoma tissues and that LDHB overexpression is associated with the development of hepatocellular carcinoma (31). The AR and LDHB genes are mostly associated with hepatocellular carcinoma, but few studies reported these genes being biomarkers for ALD. The present study is the first to reveal the significance of these genes in ALD. These hub genes could demonstrate the key target roles for GH in improving ALD. Multiple pathways are connected by shared targets, suggesting a possible synergistic effect between pathways on the preventive effect of GH against ALD. Meanwhile, the result suggests that GH has promising applications in preventing and controlling cancer, specifically in preventing the further development of ALD into cirrhosis and liver cancer.

ALD is defined as liver damage and its range of pathologies due to chronic and/or heavy alcohol consumption. Due to the complex pathological processes that follow the metabolism of alcohol in the liver, there is growing evidence that oxidative stress, endoplasmic reticulum stress, and hepatocyte regulation are all closely associated with ALD (32). Despite the increasing number of reports on ginseng functional foods, the underlying mechanism of the preventive effect of fermented ginseng on ALD *in vivo* remains unclear. The difference between GH and GSW is due to the processing of the herb and the specificity of the target action and therefore warrants further investigation. Our previous group confirmed the protective effect of ginsenoside extract on alcoholic liver injury through animal experiments. Rk3 can inhibit apoptosis to regulate alcoholic liver injury, and Rh1 and CK can significantly improve liver enzyme levels and mitochondrial structure in ALD rats when mixed in a 1:1 ratio (33). Lai et al. reported that Rb1 restores glutathione levels (34). In the present study, we proposed a hypothesis for ALD prevention by using network pharmacology and found that GH improves alcoholic liver injury by protecting against antioxidative stress. We investigated the mechanism of action of GH in ALD treatment by combining network pharmacology with gene microarray data set and provided a theoretical basis and direction for subsequent experiments to verify its exact mechanism.

## Conclusion

The effects of different processing technologies on the NVOCs of ginseng wine samples were monitored using UPLC-Q-Orbitrap-HRMS and multivariate statistical analyses. The results revealed distinct differences in NVOCs characteristics among the samples. GH had higher concentrations of rare ginsenosides and more ginsenosides. The active ingredients and targets of GH in the treatment of ALD were studied by using the method of network pharmacology. Ginsenosides may perform therapeutic activities by regulating targets, such as GSTP1, HRAS, AKR1B1, GSTA1, AR, GSR, and LDHB. Functionalization of ginseng Huang jiu will not only improve its taste and offer more freshness to the public, but also promote the future development of the Huang jiu sector by using medicine and food homologous ginseng as an auxiliary ingredient. In this network pharmacology study, a multicomponent, multitarget, and multi-pathway treatment of ALD with GH was established, providing a theoretical basis for the application of GH in ALD treatment. However, the results of this study are based on data analysis. They have only a certain predictive effect, which needs to be verified by further *in vitro* and *in vivo* experiments.

## Data availability statement

The original contributions presented in the study are included in the article/Supplementary material, further inquiries can be directed to the corresponding authors.

## Author contributions

YW: investigation, formal analysis, visualization, and writing—original draft. YC: methodology. LM: investigation. FL: formal analysis. MZ and YW: conceptualization and methodology. FZ and ZP: conceptualization and writing—review and editing. HY: project administration and funding acquisition. All authors contributed to the article and approved the submitted version.

## Funding

This study was financially supported by Scientific and Technological Development Program of Jilin Province of China (Grant Number 20200201141JC and 20210204046YY), and Postdoctoral Key Program of Jilin Province.

## Conflict of interest

The authors declare that the research was conducted in the absence of any commercial or financial relationships that could be construed as a potential conflict of interest.

## Publisher's note

All claims expressed in this article are solely those of the authors and do not necessarily represent those of their affiliated organizations, or those of the publisher, the editors and the reviewers. Any product that may be evaluated in this article, or claim that may be made by its manufacturer, is not guaranteed or endorsed by the publisher.
