# Supplementary material for: Identification and chemical profiling of anti-alcoholic liver disease biomarkers of ginseng Huang jiu using UPLC-Q-Orbitrap-HRMS and network pharmacology-based analyses
Source: Front Nutr. 2022 Aug 12;9:978122. doi: 10.3389/fnut.2022.978122 (PMC9412739; doi:10.3389/fnut.2022.978122)
Supplement: Supplementary file 1 [file Table_1.DOCX]

Supplementary Material for

Identification and chemical profiling of anti-alcoholic liver disease biomarkers of ginseng Huang jiu using UPLC-Q-Orbitrap-HRMS and network pharmacology-based analyses

**Yongxi Wu^1^, Yongyu Cai, Liting Ma, Fangtong Li, Meiyu Zhang, Yizhu Wang, Fei Zheng*, Zifeng Pi*, Hao Yue**

^1^Changchun University of Chinese Medicine, Changchun 130117, China

* Correspondence: Fei Zheng; E-mail: zhengfei@ccucm.edu.cn;

* Correspondence: Zifeng Pi; E-mail: 178521212@qq.com

**Supplementary Table 1** Identification of the NVOCs composition in Ginseng Huang jiu and Ginseng soaked wine

| **No.** | **RT (min)** | **Compound** | **[M-H]-/ [M+COOH]- Expected *m*/*z*** | **[M-H]-/ [M+COOH]- Measured *m*/*z*** | **Formula** | **Error (ppm)** | **GH** | **GSW** | **classification** |
| --- | --- | --- | --- | --- | --- | --- | --- | --- | --- |
|  | 2.25 | NotoginsenosideR3 | 1007.54330 | 1007.54351 | C_48_H_82_O_19_ | -0.2 | + | - | PPT |
|  | 2.42 | Re3 | 1007.54330 | 1007.54361 | C_48_H_82_O_19_ | -0.3 | + | + | PPT |
|  | 2.97 | NotoginsenosideM | 1077.54512 | 1077.54331 | C_48_H_82_O_19_ | 1.8 | + | - | PPT |
|  | 4.33 | (O+PPT)-Glc-Glc | 861.48535 | 861.48735 | C_42_H_72_O_15_ | -2 | + | + | PPT |
|  | 5.00 | (PPT+H_2_O)-Glc-(Glc-Rha) | 1009.55831 | 1009.56051 | C_48_H_78_O_19_ | -2.2 | + | + | PPT |
|  | 5.65 | 20-*O*-Glc-Rf | 1007.54330 | 1007.54330 | C_48_H_82_O_19_ | 0 | + | + | PPT |
|  | 6.39 | (O+PPT)-Glc-Glc | 861.48535 | 861.48825 | C_42_H_72_O_15_ | -2.9 | + | + | PPT |
|  | 7.67 | PPT-Glc-Glc-Glc | 1007.54330 | 1007.54362 | C_48_H_82_O_19_ | -0.3 | + | + | PPT |
|  | 8.56 | 20(S)-Rf2 | 801.50062 | 801.49990 | C_42_H_74_O_14_ | 0.7 | + | + | PPT |
|  | 9.18 | VinaginsenosideR4 | 1007.54330 | 1007.54321 | C_48_H_82_O_19_ | 0.1 | + | + | PPT |
|  | 9.38 | NotoginsenosideR1 | 977.52855 | 977.53282 | C_47_H_80_O_18_ | -4.3 | + | + | PPT |
|  | 9.59 | 20(R)-Rf2 | 801.50062 | 801.49910 | C_42_H_74_O_14_ | 1.5 | + | - | PPT |
|  | 10.31 | Re4 | 977.52855 | 977.53322 | C_47_H_80_O_18_ | -4.7 | + | + | PPT |
|  | 12.99 | Rg1 | 845.49050 | 845.49031 | C_42_H_72_O_14_ | 0.2 | + | + | PPT |
|  | 13.51 | Re | 991.54833 | 991.54823 | C_48_H_82_O_18_ | 0.1 | + | + | PPT |
|  | 14.59 | Pseudo-ginsenoside F11 or isomer | 845.49050 | 845.49131 | C_42_H_72_O_14_ | -0.8 | + | + | Unknown |
|  | 15.02 | (BB-1/2)-(Glc-Glc)-(Glc-Xyl) | 1157.59581 | 1157.59881 | C_53_H_92_O_24_ | -3 | + | + | Unknown |
|  | 15.20 | mRg1 | 885.48537 | 885.48597 | C_45_H_74_O_17_ | -0.6 | + | + | PPT |
|  | 15.61 | mRe | 1031.54334 | 1031.54414 | C_51_H_84_O_21_ | -0.8 | + | + | PPT |
|  | 16.22 | mRf | 885.48537 | 885.48627 | C_45_H_74_O_17_ | -0.9 | + | + | PPT |
|  | 16.61 | PPT-Glc-Glc-Glc-Glc | 1169.59500 | 1169.59880 | C_54_H_92_O_24_ | -3.8 | + | + | PPT |
| **No.** | **RT (min)** | **Compound** | **[M-H]-/ [M+COOH]- Expected *m*/*z*** | **[M-H]-/ [M+COOH]- Measured *m*/*z*** | **Formula** | **Error (ppm)** | **GH** | **GSW** | **classification** |
|  | 16.67 | noto-Fc or isomer | 1209.59130 | 1209.59340 | C_58_H_98_O_26_ | -2.1 | + | + | PPD |
|  | 16.80 | 508-(Glc-Glc)-(Glc-Xyl) | 1171.57493 | 1171.57333 | C_54_H_94_O_24_ | 1.6 | + | + | Unknown |
|  | 16.97 | 20(S)-Rf3 | 817.49556 | 817.49536 | C_42_H_74_O_15_ | 0.2 | + | - | PPT |
|  | 17.10 | noto-Fc or isomer | 1209.59130 | 1209.59340 | C_58_H_98_O_26_ | -2.1 | + | + | PPD |
|  | 17.21 | Re5 | 815.47922 | 815.48052 | C_42_H_72_O_15_ | -1.3 | + | + | PPT |
|  | 17.48 | Isomer of Rg2 | 829.49550 | 829.49730 | C_42_H_72_O_13_ | -1.8 | + | - | PPT |
|  | 17.56 | F3 | 815.47922 | 815.48042 | C_41_H_70_O_13_ | -1.2 | + | + | PPT |
|  | 18.01 | Ginsenoside La | 829.49550 | 829.49730 | C_42_H_72_O_13_ | -1.8 | + | + | PPT |
|  | 18.38 | floral-A/floral-B | 831.47112 | 831.47662 | C_42_H_72_O_16_ | -5.5 | + | + | Unknown |
|  | 18.60 | Rf | 845.49050 | 845.49041 | C_42_H_72_O_14_ | 0.1 | + | + | PPT |
|  | 19.30 | chikusetsusaponin Fk1 or isomer | 945.47111 | 945.47190 | C_48_H_82_O_18_ | -0.8 | + | - | Unknown |
|  | 19.53 | NotoginsenosideR2 | 815.47922 | 815.48152 | C_41_H_70_O_13_ | -2.3 | + | + | PPT |
|  | 19.93 | Isomer of mRg1 | 885.48537 | 885.48727 | C_45_H_74_O_17_ | -1.9 | + | + | PPT |
|  | 20.00 | NotoginsenosideR4 | 1285.64353 | 1285.64492 | C_59_H_100_O_27_ | -1.4 | + | + | PPT |
|  | 20.15 | (PPT+H_2_O)-Glc-(Glc-Rha) | 1009.55831 | 1009.56071 | C_48_H_78_O_19_ | -2.4 | + | + | PPT |
|  | 20.68 | F5 | 815.47922 | 815.48052 | C_41_H_70_O_13_ | -1.3 | + | - | PPT |
|  | 20.93 | 20(S)Rh1 | 683.43700 | 683.43731 | C_36_H_62_O_9_ | -0.3 | + | - | PPT |
|  | 21.04 | 20(S)Rg2 | 829.49550 | 829.49711 | C_42_H_72_O_13_ | -1.6 | + | + | PPT |
|  | 21.35 | 20(R)Rg2 | 829.49550 | 829.49710 | C_42_H_72_O_13_ | -1.6 | + | + | PPT |
|  | 21.87 | 20(R)Rh1 | 683.43700 | 683.43792 | C_36_H_62_O_9_ | -0.9 | + | + | PPT |
|  | 22.09 | Ra3 | 1285.64353 | 1285.64603 | C_59_H_100_O_27_ | -2.5 | + | + | PPD |
|  | 22.56 | Ra2 | 1255.63281 | 1255.63321 | C_58_H_97_O_26_ | -0.4 | + | + | PPD |
|  | 23.00 | Rb1 | 1153.59722 | 1153.60014 | C_54_H_92_O_23_ | -2.9 | + | + | PPD |
|  | 23.69 | mRa2 | 1295.62782 | 1295.62901 | C_61_H_100_O_29_ | -1.2 | + | + | PPD |
|  | 23.98 | Ro | 955.49561 | 955.49561 | C_48_H_76_O_19_ | 0 | + | + | OLE |
|  | 24.09 | mRb1 | 1193.59621 | 1193.59630 | C_57_H_94_O_26_ | -0.1 | + | + | PPD |
|  | 24.32 | Rc | 1123.59077 | 1123.59127 | C_53_H_90_O_22_ | -0.5 | + | + | PPD |
|  | 24.42 | Compound Y1 | 847.50616 | 847.50746 | C_42_H_74_O_14_ | -1.3 | + | + | PPD |
|  | 24.72 | Ra1 | 1255.63281 | 1255.63510 | C_58_H_97_O_26_ | -2.3 | + | + | PPD |
|  | 25.16 | Compound Y2 | 847.50616 | 847.50716 | C_42_H_74_O_14_ | -1 | + | - | PPD |
|  | 25.38 | mRc | 1163.58552 | 1163.58712 | C_56_H_92_O_25_ | -1.6 | + | + | PPD |
|  | 25.55 | mRa1 | 1295.62782 | 1295.62901 | C_61_H_100_O_29_ | -1.2 | + | + | PPD |
|  | 25.63 | Rb2 | 1123.59077 | 1123.59127 | C_53_H_90_O_22_ | -0.5 | + | + | PPD |
|  | 26.00 | Rb3 | 1123.59077 | 1123.59127 | C_53_H_90_O_22_ | -0.5 | + | + | PPD |
|  | 26.35 | mRb2 | 1163.58552 | 1163.58712 | C_56_H_92_O_25_ | -1.6 | + | + | PPD |
|  | 26.63 | mRb3 | 1163.58552 | 1163.58712 | C_56_H_92_O_25_ | -1.6 | + | + | PPD |
|  | 26.96 | ChikusetsusaponinⅣa | 793.43783 | 793.43913 | C_42_H_66_O_14_ | -1.3 | + | + | OLE |
| **No.** | **RT (min)** | **Compound** | **[M-H]-/ [M+COOH]- Expected *m*/*z*** | **[M-H]-/ [M+COOH]- Measured *m*/*z*** | **Formula** | **Error (ppm)** | **GH** | **GSW** | **classification** |
|  | 27.28 | Rd | 991.54833 | 991.54863 | C_48_H_82_O_18_ | -0.3 | + | + | PPD |
|  | 27.44 | NotoginsenosideK | 991.54833 | 991.54793 | C_48_H_82_O_18_ | 0.4 | + | + | PPD |
|  | 27.82 | mRd | 1031.54334 | 1031.54574 | C_51_H_84_O_21_ | -2.4 | + | + | PPD |
|  | 28.32 | Rs2 | 1119.59570 | 1119.59470 | C_55_H_92_O_23_ | 1 | + | - | PPD |
|  | 28.47 | Gypenoside XVII | 991.54833 | 991.54833 | C_48_H_82_O_18_ | 0 | + | + | PPD |
|  | 28.50 | Elatoside K | 1117.54382 | 1117.54501 | C_54_H_86_O_24_ | -1.2 | + | + | OLE |
|  | 28.79 | Isomer of mRd | 1031.54334 | 1031.54574 | C_51_H_84_O_21_ | -2.4 | + | + | PPD |
|  | 28.99 | floral-P/floral-O | 1093.58001 | 1093.58261 | C_53_H_90_O_23_ | -2.6 | + | + | Unknown |
|  | 29.39 | NotoginsenosideFe | 961.53782 | 961.54022 | C_47_H_80_O_17_ | -2.4 | + | + | PPD |
|  | 29.69 | Gypenoside IX | 961.53782 | 961.54033 | C_47_H_80_O_17_ | -2.5 | + | + | PPD |
|  | 30.08 | Compound O | 961.53782 | 961.54022 | C_47_H_80_O_17_ | -2.4 | + | + | PPD |
|  | 30.36 | Notoginsenoside Fd | 961.53782 | 961.54022 | C_47_H_80_O_17_ | -2.4 | + | + | PPD |
|  | 30.57 | Rg6 | 811.48491 | 811.48540 | C_42_H_70_O_12_ | -0.5 | + | + | PPT |
|  | 30.66 | Rg4 | 811.48491 | 811.48540 | C_42_H_70_O_12_ | -0.5 | + | + | PPT |
|  | 31.29 | F4 | 811.48491 | 811.48540 | C_42_H_70_O_12_ | -0.5 | + | + | PPT |
|  | 31.40 | Rk3 | 665.42709 | 665.42698 | C_36_H_60_O_8_ | 0.1 | + | + | PPT |
|  | 31.99 | Rh4 | 665.42709 | 665.42758 | C_36_H_60_O_8_ | -0.5 | + | + | PPT |
|  | 32.18 | F2 | 829.49550 | 829.49573 | C_42_H_72_O_13_ | -0.2 | + | + | PPD |
|  | 32.65 | 458-(Glc-Glc) | 827.47983 | 827.48193 | C_42_H_70_O_13_ | -2.1 | + | + | Unknown |
|  | 32.89 | Zingibroside R1 | 793.43783 | 793.43883 | C_42_H_66_O_14_ | -1 | + | + | OLE |
|  | 33.16 | Rg9 | 827.47983 | 827.48143 | C_42_H_70_O_13_ | -1.6 | + | - | PPT |
|  | 33.31 | Rg8 | 827.47983 | 827.48143 | C_42_H_70_O_13_ | -1.6 | + | - | PPT |
|  | 33.91 | Gypenoside LXXV | 829.49550 | 829.49573 | C_42_H_72_O_13_ | -0.2 | + | + | PPD |
|  | 34.19 | Rg3 | 829.49550 | 829.49681 | C_42_H_72_O_13_ | -1.3 | + | + | PPD |
|  | 34.23 | 468-Glc-Rha-Glc | 937.48140 | 937.48012 | C_44_H_74_O_21_ | 1.3 | + | + | Unknown |
|  | 34.76 | notoginsenosideRw1 | 901.48515 | 901.48597 | C_46_H_78_O_17_ | -0.8 | + | - | PPT |
|  | 34.87 | 20(S)Rs3 | 825.50060 | 825.49980 | C_44_H_74_O_14_ | 0.8 | + | + | PPD |
|  | 35.05 | chikusetsusaponin L5 | 901.48515 | 901.48616 | C_46_H_78_O_17_ | -1 | + | - | PPT |
|  | 35.12 | 20(R)Rs3 | 825.50060 | 825.49991 | C_44_H_74_O_14_ | 0.7 | + | + | PPD |
|  | 36.56 | Rk1 | 811.48491 | 811.48512 | C_42_H_70_O_12_ | -0.2 | + | + | PPD |
|  | 36.79 | Rg5 | 811.48491 | 811.48530 | C_42_H_70_O_12_ | -0.4 | + | + | PPD |
|  | 36.97 | Rs4 | 807.49004 | 807.48961 | C_44_H_72_O_13_ | 0.4 | + | + | PPT |
|  | 37.19 | Rh2 | 667.44271 | 667.44291 | C_36_H_62_O_8_ | -0.2 | + | + | PPD |
|  | 37.34 | CK | 667.44271 | 667.44303 | C_36_H_62_O_8_ | -0.3 | + | + | PPD |
|  | 39.53 | 20(S)Rh3 | 649.43211 | 649.43330 | C_36_H_60_O_7_ | -1.2 | + | - | PPT |
|  | 39.71 | 20(R)Rh3 | 649.43211 | 649.43321 | C_36_H_60_O_7_ | -1.1 | + | - | PPT |
|  | 41.17 | PPT | 521.38482 | 521.38212 | C_30_H_52_O_4_ | 2.7 | + | - | PPT |
|  | 42.31 | PPD | 459.38442 | 459.38142 | C_30_H_52_O_3_ | 3 | + | - | PPD |

Note: “+” means there had detectable. “-” means there had no detectable.

**Supplementary Table 2** Scoring table with different algorithms for 41 genes

| name | MCC | Degree | EPC | EcCentricity | Closeness | Betweenness |
| --- | --- | --- | --- | --- | --- | --- |
| THRB | 2 | 2 | 4.449 | 0.2 | 17.53333 | 2.62404 |
| PMS2 | 1 | 1 | 3.574 | 0.2 | 16.36667 | 0 |
| PNP | 1 | 1 | 2.099 | 0.25 | 14.08333 | 0 |
| HEXB | 1 | 1 | 1.328 | 0.2 | 10.98333 | 0 |
| GM2A | 2 | 2 | 2.64 | 0.25 | 14.75 | 78 |
| GALK1 | 2 | 2 | 3.093 | 0.2 | 14.53333 | 1 |
| FGFR2 | 40320 | 8 | 14.504 | 0.25 | 21.91667 | 0 |
| CTSK | 7 | 4 | 8.479 | 0.2 | 19.2 | 3.61905 |
| BTK | 120 | 5 | 11.526 | 0.25 | 19.25 | 0 |
| STS | 2 | 2 | 5.899 | 0.2 | 16.45 | 0 |
| F2 | 68 | 10 | 13.015 | 0.25 | 23.91667 | 45.13871 |
| IGF1 | 48654 | 21 | 17.574 | 0.25 | 29.91667 | 185.0624 |
| ELANE | 4 | 3 | 6.968 | 0.25 | 19.08333 | 0.66667 |
| F11 | 30 | 5 | 7.675 | 0.25 | 20.25 | 2.2386 |
| MMP1 | 56 | 7 | 11.74 | 0.25 | 21.91667 | 13.93785 |
| MET | 46968 | 13 | 16.706 | 0.25 | 25.41667 | 23.99119 |
| F10 | 24 | 4 | 7.537 | 0.25 | 19.58333 | 0 |
| HPRT1 | 6 | 5 | 8.038 | 0.33333 | 21.33333 | 226.8 |
| MAP2K1 | 1584 | 9 | 14.872 | 0.25 | 23.58333 | 7.2782 |
| GBA | 2 | 2 | 4.492 | 0.25 | 18.58333 | 20.68 |
| TGFB2 | 30 | 5 | 10.231 | 0.25 | 20.58333 | 1.06667 |
| TGFBR1 | 18 | 5 | 10.786 | 0.25 | 21.25 | 3.02053 |
| GP1BA | 25 | 5 | 9.196 | 0.25 | 20.91667 | 8.59298 |
| CA2 | 3 | 3 | 6.518 | 0.25 | 19.58333 | 6.94951 |
| NR3C1 | 72 | 7 | 13.016 | 0.25 | 22.58333 | 11.62941 |
| GCK | 25 | 5 | 9.744 | 0.25 | 20.91667 | 55.32 |
| ALB | 6784 | 25 | 17.918 | 0.33333 | 32.33333 | 595.6015 |
| PAH | 14 | 5 | 7.893 | 0.25 | 20.25 | 28.3337 |
| TTR | 15 | 6 | 9.036 | 0.25 | 21.41667 | 74.93635 |
| AGXT | 2 | 2 | 3.69 | 0.2 | 14.7 | 0 |
| PPARG | 147 | 9 | 13.605 | 0.33333 | 23.83333 | 112.7942 |
| ACADM | 1 | 1 | 3.385 | 0.25 | 15 | 0 |
| PIK3R1 | 42896 | 17 | 17.011 | 0.25 | 26.91667 | 101.5965 |
| HRAS | 49501 | 18 | 17.62 | 0.25 | 28.08333 | 143.18 |
| ESR1 | 49160 | 16 | 17.044 | 0.25 | 26.91667 | 86.94015 |
| JAK2 | 47760 | 13 | 16.575 | 0.25 | 25.58333 | 26.06045 |
| AR | 1610 | 10 | 15.091 | 0.25 | 23.91667 | 36.57294 |
| PTPN11 | 47784 | 13 | 16.348 | 0.25 | 25.41667 | 21.39214 |
| CTNNA1 | 145 | 7 | 12.967 | 0.2 | 21.03333 | 12.29531 |
| KIT | 47762 | 13 | 16.462 | 0.33333 | 25.83333 | 87.80201 |
| ABL1 | 864 | 8 | 13.89 | 0.25 | 21.41667 | 2.87902 |
